# Flavor and sensory profile of Chinese traditional fish noodles produced by different silver carp (*hypophthalmichthys molitrix*) mince ingredients

**DOI:** 10.1016/j.fochx.2023.100977

**Published:** 2023-11-01

**Authors:** Hongyu Zhou, Zhiwei Hu, Youming Liu, Shanbai Xiong

**Affiliations:** a College of Food Science and Technology/National R&D Branch Center for Conventional Freshwater Fish Processing (Wuhan), Huazhong Agricultural University, Wuhan, Hubei Province 430070, PR China; b Engineering Research Center of Green Development for Conventional Aquatic Biological Industry in the Yangtze River Economic Belt, Ministry of Education, Wuhan, Hubei Province 430070, PR China

**Keywords:** Fish noodle, Volatile compounds, Sensory lexicon, GC-IMS, GC-MS

## Abstract

This study employs sensory evaluation, headspace gas chromatography–ion mobility spectrometry (HS-GC-IMS), and headspace solid-phase microextraction gas chromatography–mass spectrometry (HS-SPME-GC-MS) techniques to investigate the effect of different pretreatment of fresh silver carp mince (running water rinsing 0, 1, or 2 times) and commercially frozen surimi on the odor characteristics of fish noodles. The free choice profiling (FCP) and check all that apply (CATA) sensory analysis methods were utilized to identify 10 characteristic descriptors, which include “grass, fish fragrance, unpleasant fishy, fatty, roast, ammonia, caramel, warmed-over, earthy, and mushroomy”. HS-GC-IMS and HS-SPME-GC-MS detected 80 and 37 volatile compounds (VCs) in fish noodles. The 1-Penten-3-ol, (E)-2-pentenal-D, hexanal-D, pentanal-D, (E,E)-2, 4-heptadienal-D contents were significantly correlated with “fish fragrance” and “unpleasant fishy”, and octanal, nonanal, heptanal, 2-methylpyrazine contents were significantly correlated with “warmed-over” flavor. The results of this study can be helpful for fish noodle quality improvement and industrial production.

## Highlights

• A sensory lexicon of fish noodle was established and 10 odor attributes were screened.
• The flavor fingerprint of fish noodles was established using GC-IMS and GC-MS.
• FS-fish noodle differed FR-fish noodle significantly in aroma profiles.
• 2-Methylpyrazine, heptanal, octanal and, nonanal contributed to “warmed-over” odor.

## 1 Introduction

Fish noodles, a traditional Chinese surimi product, have been consumed for over a thousand years and were designated as a Chinese Geographical Indication product in 2010. Due to its long history, unique flavor, and rich nutrition, it is a special food and an important part of the daily diet in the Hubei and Fujian provinces. Traditional fish noodles are made from fresh silver carp mince and sweet potato starch through chopping, mixing, plasticizing, steam heating, slicing, and drying. Generally, fish noodles produced in the Hubei Province are cooked, while those produced in the Fujian province are raw. Currently, there are many brands of fish noodles available on the market, but most of them are produced in small family workshops. The low production capacity and varying quality have seriously hindered the development of the fish noodle industry. However, large-scale production will inevitably become the direction of fish noodle development.

Frozen surimi is made from live fish by harvesting, filtering, rinsing, dewatering, refining, and freezing. Compared with fresh fish mince, frozen surimi has the advantages of low price, storage resistance, and stable quality and is the main raw material for most surimi products in the market (fish balls, kamaboko, and crablike sticks). Nevertheless, studies have revealed that during freezing and storage of surimi, protein denaturation, lipid oxidation, non-enzymatic rancidity, and chemical changes caused by microbial metabolism deteriorate the texture and flavor quality of surimi and its products. Specifically, during freezing, flavor precursors such as free amino acids, fatty acids, and nucleotides in surimi are degraded, further affecting the flavor formation of surimi products during thermal processing (Kosowska et al., 2017). Flavor quality is one of the important qualities of surimi and its products, directly affecting the consumers' choice of products (Sohail et al., 2022).

Rinsing, as an important pretreatment in the processing of surimi and its products, reduces the flavor compounds and certain flavor-related precursors and changes its flavor characteristics. Different rinsing methods affect flavor characteristics differently (Zhou et al., 2016). Additionally, our research team found in previous work that rinsing changed the chemical composition of surimi and investigated the interplay between surimi chemical composition and the flavor quality of surimi gel (Li et al., 2023). However, the effect of rinsed fresh fish mince and frozen surimi on fish noodles' flavor profile is unknown.

Current methods analyzing the aroma characteristics in food products are mainly sensory and instrumental (Chen et al., 2021). Among the many sensory analysis methods, the FCP method requires sensory personnel to describe the product freely, generating descriptors without requirements for the number and type of descriptors. The CATA method is a form of multiple choice survey that provides sensory personnel with a list of alternative answers, which check off the options they believe apply to the product. Lazo et al. (2016) used FCP and CATA methods to assess the flavor and texture of nine fish species. Nie et al. (2023) used FCP and CATA methods to create a sensory dictionary with 24 monascus purpureus fermented kelp descriptors. Among the instrumental analyses, HS-SPME-GC-MS is a highly efficient food aroma analysis technique widely used for separating, characterizing, and quantifying volatile compounds (VCs) in food (Chen et al., 2021). VCs in analytes are separated by intensity differences in interactions with the stationary phase and are identified by the mass spectrometer by compound “fingerprint” information. However, the low selectivity, complex pre-processing, and long detection times of GC-MS technology for complex food samples inevitably affect the accuracy and timeliness of sample detection. HS-GC-IMS is an emerging technique to analyze VCs, which combines the high separation capability of gas chromatography with the fast response capability of ion mobility spectrometry, making it highly sensitive, allowing rapid analysis and visualization for food flavor detection (Feng et al., 2022, Wang et al., 2019). However, the incomplete database of the GC-IMS technique remains a non-negligible drawback, limiting the complete characterization and accurate quantification of VCs in samples (Liu et al., 2023, Lu et al., 2022). Therefore, a combined HS-SPME-GC-MS and HS-GC-IMS approach for detecting VCs in samples may be an ideal strategy for more comprehensive information on volatiles.

In this study, fish noodles were prepared from fresh fish mince (rinsing 0, 1, or 2 times) and frozen surimi. The objectives of this study were: (1) Establish a sensory dictionary for evaluating fish noodles using FCP and CATA methods, (2) Establish volatile fingerprint profiles of fish noodles by GC-IMS and HS-SPME-GC-MS, (3) Explore the relationship between sensory attributes and flavor substances of fish noodles by multivariate statistical analysis, and (4) Investigate the effect fresh and frozen fish mince on the aroma characteristics of fish noodles. This study has important theoretical and practical implications for guiding the industrial production of fish noodles.

## 2 Materials and methods

### 2.1 Chemicals

The *n*-ketones (C4-C9) with analytical reagent grade were purchased from Sinopharm Chemical Reagent (Shanghai, China). Hexanal (99 %), heptanal (≥98 %), octanal (96 %), (E)-2-octenal (95 %), nonanal (96 %), decanal (97 %), (E,E)-2,4-Heptadienal (>90 %), 1-octen-3-ol (>98 %), 1-hexanol (>95 %), 1-decanol (>95 %), and 1-penten-3-ol (>95 %) were purchased from Aladdin Biochemical Technology Co.,Ltd. (Shanghai, China). Cyclohexanone (>99.8 %), and *n*-alkane (C6-C26) were chromatographic grade reagent purchased from Sigma-Aldrich (St. Louis, MO, U.S.A.). Other chemicals were analytical grade and were purchased from China Pharmaceutical Chemical Reagent Co, Ltd. (Shanghai, China).

### 2.2 Materials

Ten fresh silver carp (*Hypophthalmichthys molitrix*) weighing 2500–3500 g were procured in winter from the vegetable market of Huazhong Agricultural University. All the fish were placed in a rectangular plastic tank with plenty of oxygen and water, and the fish were transported to the lab alive in 30 min using a car. Frozen surimi (Grade AAA) was purchased from Jingli Aquaculture Company (Honghu, Hubei, China), commercially prepared from silver carp. Sweet potato (968-19) starch was purchased from Hubei Longzhiquan Agricultural Development Company (Danyang, Hubei, China).

### 2.3 Sample preparation

#### 2.3.1 Fresh fish mince and frozen surimi

A physical blow to the head killed live silver carp and then beheaded, gutted, and washed, following the Guidance on Treating Experimental Animals developed by China’s Ministry of Science & Technology in 2006 and Regulations issued by China State Council in 1988. The cleaned fish was put into the meat separator (HZ250, Yingbo Machinery Co., Ltd., Xiamen, Fujian), and then the skin and bones were separated, and the minced fish was collected. Fish mince was divided into three groups for rinsing: the first group of fish mince was not rinsed, the second group was rinsed once, and the third group was rinsed twice. Rinsing: the same mass of fish meat and crushed ice was put into the rinsing bucket, and tap water of three times the mass of fish mince was added, stirred, and rinsed for 15 min. Then, the fish mince was filtered using a strainer, followed by centrifugation at 3000 r/min for 10 min using a dehydrator to make the water content lower than 78 % and packed in vacuum bags with about 500 g per bag. The fish mince was stored at 4 °C and ran out on the day of the experiment.

Frozen surimi was removed from the cold storage at −20 °C 12 h before the start of the experiment and thawed in a refrigerator at 4 °C to be used. Additionally, the moisture content of the surimi was controlled below 78 % by centrifugation.

#### 2.3.2 Fish noodles

The fresh fish mince/ frozen surimi was first chopped using a food processor (FP-3010) for one minute. Then, the fresh fish mince/ frozen surimi was mixed with 2.5 % (w/w) salt and chopped for three minutes. Then, the fish mince was mixed with 100 % (w/w) sweet potato starch and 8 % (w/w) water to the mixer (JM-7LG) and mixed for 10 min. The dough was removed, and a rolling pin was used to change the shape of the dough into a disc shape with a diameter of 250 ± 50 mm and a thickness of 1.5 ± 0.3 mm. After that, it was put into the steamer for 15 min, removed, and rolled. When cooled to room temperature, it was cut into thin slices with a thickness of 1.5 ± 0.3 mm, put in a 50 °C hot air drying oven (DHG-9240A), and dried with hot air for 3 h. Then, the fish noodles were obtained.

FR-fish noodles represented fish noodles made from fresh fish minced after rinsing. FR0, FR1, and FR2 represented the fresh fish mince with 0, 1, and 2 rinsing times, respectively. FS-fish noodles represented fish noodles made from frozen surimi.

### 2.4 Sensory analysis

#### 2.4.1 Establishment of sensory lexicon

The sensory evaluation was conducted according to Li et al. (2022). The sensory panel comprised 15 evaluators (8 males and 7 females) from the Freshwater Product Processing Theory and Technology Innovation Team of Huazhong Agricultural University with extensive sensory experience in aquatic products for between 22 and 40 years. The FCP method was first used, asking the visitors to generate sensory descriptors to describe their perceived odors freely. This was followed by the CATA method, which provided the sensory panel with a list of 25 sensory descriptors. Before the CATA test, the sensory group members agreed on the 25 sensory descriptor definitions. Each experiment was implemented twice, and the quoting frequency (%) was determined by the occurred time with the ratio of total test times (30).

#### 2.4.2 Quantitative descriptive analysis

The quantitative descriptive sensory and overall flavor scoring tests adopted the ISO 10399:2017 methodology. The scoring settings were proposed by Li et al. (2022).

### 2.5 HS-GC-IMS analysis

The VCs in fish noodle samples were analyzed by GC-IMS equipment (FlavourSpec®, g.a.s. Dortmund, Germany) using the method of Luo et al. (2022). A 2 g fish noodle sample was placed in a 20 mL headspace vial, sealed, and incubated at 60 °C for 20 min. Then, 500 μL of sample gas was taken as headspace injection into a wax column (ID: 0.53 mm, film thickness 1 μm RESTEK, USA). The injection needle temperature was kept at 85 °C, and the incubation speed was 500 rpm. Nitrogen was used as the carrier gas, and the program flow rate was set to 2 mL/min (10 min), 10 mL/min (10 min), 100 mL/min (10 min), and stop. The drift gas flow rate was set to a constant 150 mL/min flow rate. The compounds were separated on a 60 °C column and then ionized at 45 °C.

The retention index (RI) was calculated by a mixture of *n*-ketones (C4-C9). VCs were identified based on drift times (Dt) and RI using standards from the National Institute of Standards and Technology (NIST) and IMS databases in the instrument software. All the measurements of the fish noodle samples were operated in three biological replicates.

### 2.6 HS-SPME-GC-MS analysis

The experiment used crushed fish noodle samples (2.0 g) placed into a 30 mL headspace vial. One micro-liter of cyclohexanone (1000 μg/kg) was added as an internal standard, adding 8 mL of saturated sodium chloride solution. The mixture was then equilibrated at 60 °C for 20 min. An activated DVB/CAR/PDMS SPME extraction head was utilized for headspace sorption for 40 min. The GC column was a DB-Wax capillary column (60 m × 0.32 mm ID × 0.25 μm film thickness, Agilent Technologies, Inc.). The GC-MS conditions were consistent with An et al. (2022b).

Compounds were positively identified by comparing mass spectra and RIs of the standards obtained in the laboratory or tentatively identified if the RIs were obtained from the literature. The RIs were calculated using a mixture of *n*-alkanes (C6-C24), calculated with reference to Li et al. (2023). The relative contents (μg/kg) of VCs were determined by multiplying the internal standard concentration by the ratio of the peak area of the VC and the internal standard peak area. It should be noted that all the above measurements of fish noodle samples were operated in three biological replicates.

### 2.7 Statistical analysis

The results were expressed as “mean ± standard deviation”. Principal component analysis (PCA) was performed using Origin 2021 (Origin-Lab, Northampton, MA). Additionally, a one-way analysis of variance (ANOVA) was performed using SPSS25 software (IBM, Armonk, NY, USA), and differences were considered statistically significant for *p* values less than 0.05. The partial least squares discriminant analysis was performed using SIMCA 14.1 (Umetrics, Malmo, Sweden), and the partial least squares regression (PLSR) was performed using Unscrambler version X10.4 (CAMO ASA, Oslo, Norway).

## 3 Results and discussion

### 3.1 Sensory evaluation of fish noodles

#### 3.1.1 Sensory lexicon and attributes determination

**Fig. 1:**
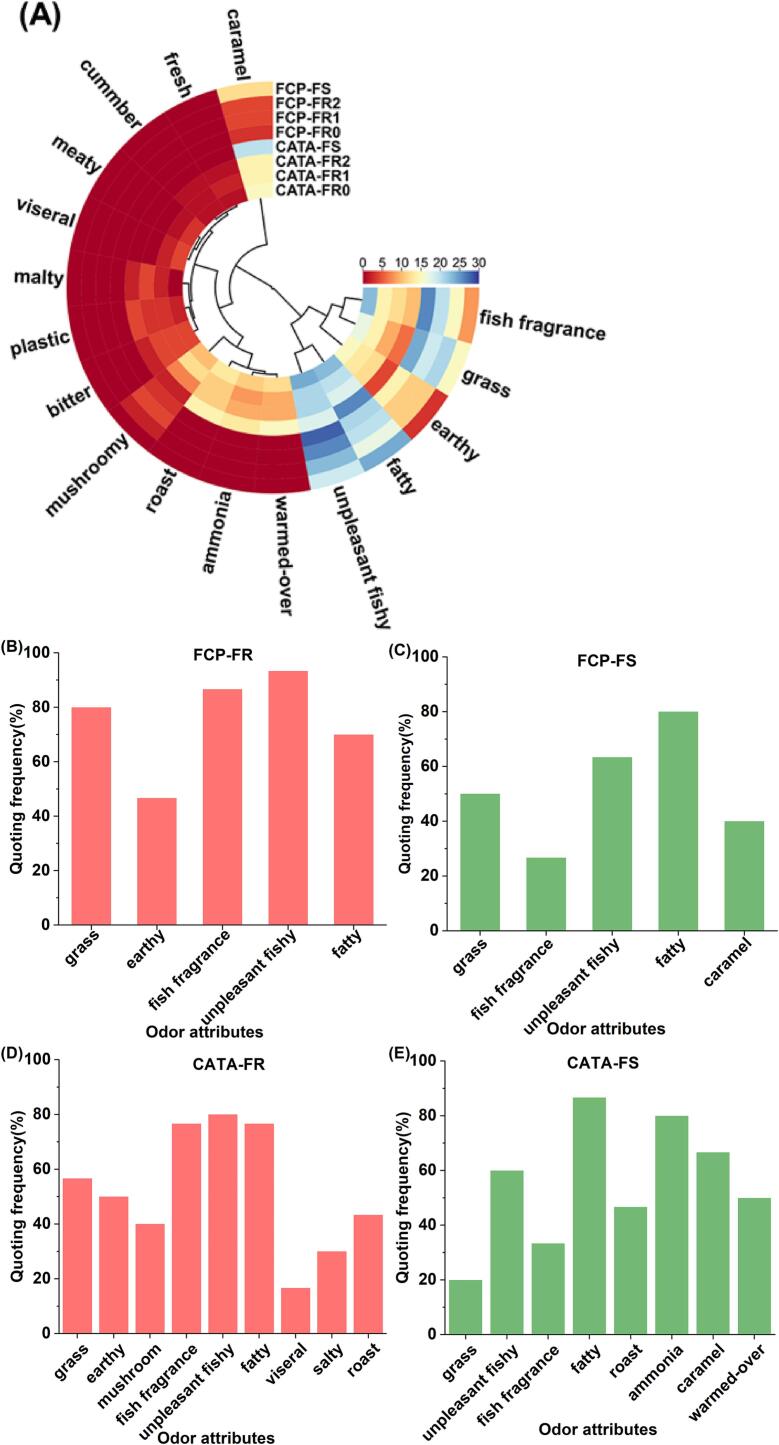
The odor sensory lexicon of fish noodle. (A) Heatmap of FCP and CATA method descriptors. (B&C) FCP descriptor for FR-fish noodle and FS-fish noodle (citation frequency > 15 %). (D&E) CATA descriptor for FR-fish noodle and FS-fish noodle (citation frequency > 15 %).

In this study, the odor-sensory evaluation of four fish noodles was conducted successively using the FCP and CATA sensory methods, with Fig. 1(A) presenting the descriptors of the samples and their citation frequencies. A total of 17 descriptors were utilized in the sensory description of the fish noodles, with 6 and 17 descriptors referenced to FCP and CATA for the FR-fish noodle samples, respectively, and 5 and 14 descriptors for the FS-fish noodle samples. Based on ISO 13299:2016, descriptors with a reference frequency > 15 % were considered odor attributes.

As illustrated in Fig. 1(B), “grass, fish fragrance, unpleasant fishy, fatty, roast, ammonia, caramel, and warmed-over” were regarded as common odor attributes of FS-fish and FR-fish noodles. The “Earthy”, “mushroomy”, “visceral”, and “salty” were the only odor attributes of the FR-fish noodles. Regarding these characteristic flavors, “unpleasant fishy” was a frequent undesirable odor in freshwater fish products, associated with trimethylamine and short-chain aldehydes, such as (E,E)-2,4-decadienal. “Grass” was derived from short-chain aldehydes, with hexanal being the representative compound. In contrast, “fatty” was derived from long-chain aldehydes, such as (E)-2-decenal. Additionally, some short-chain aldehydes with a green odor have been reported to exhibit a “greasy and fatty” odor at high concentrations, such as nonanal. The noteworthy “warmed-over” flavor is an important odor attribute affecting the odor quality of surimi products, related to the quality of the raw material and processing temperature. It is often described as an unpleasant cardboard-like, sour, or unfresh flavor (An et al., 2022a). Other odors were often defined as flavor characteristics in fish products, such as strong “fish fragrance” and “caramel” odor in surimi gel from silver carp (Li et al., 2023), slight “ammonia” odor in sturgeon meat (Li et al., 2022), and a distinct “mushroom” and “earthy” odor in grass carp mince (Xiao et al., 2022).

Discussions with sensory personnel revealed that experienced aquatic product sensory personnel still forgot some descriptors during the sensory process. The number of descriptors collected by the FCP sensory method was low, but the descriptors collected were often intuitive odor attributes of the product. Therefore, a training session for sensory users to define the descriptors after the FCP test was crucial for the subsequent CATA test. In the CATA test, the descriptors in the evaluation form helped the sensitizers quickly match sample odors to descriptors. A substantial number of odor attributes undescribed in the FCP were referenced, enriching the sensory vocabulary of the product. Therefore, for a new object, the synergistic employment of FCP and CATA methods to accurately match suitable descriptors with sample odor attributes could contribute to better establishing a sensory lexicon for the product. This study established an exclusively sensory lexicon for fish noodles to improve quality and standardized production.

#### 3.1.2 Overall odor evaluation

**Fig. 2:**
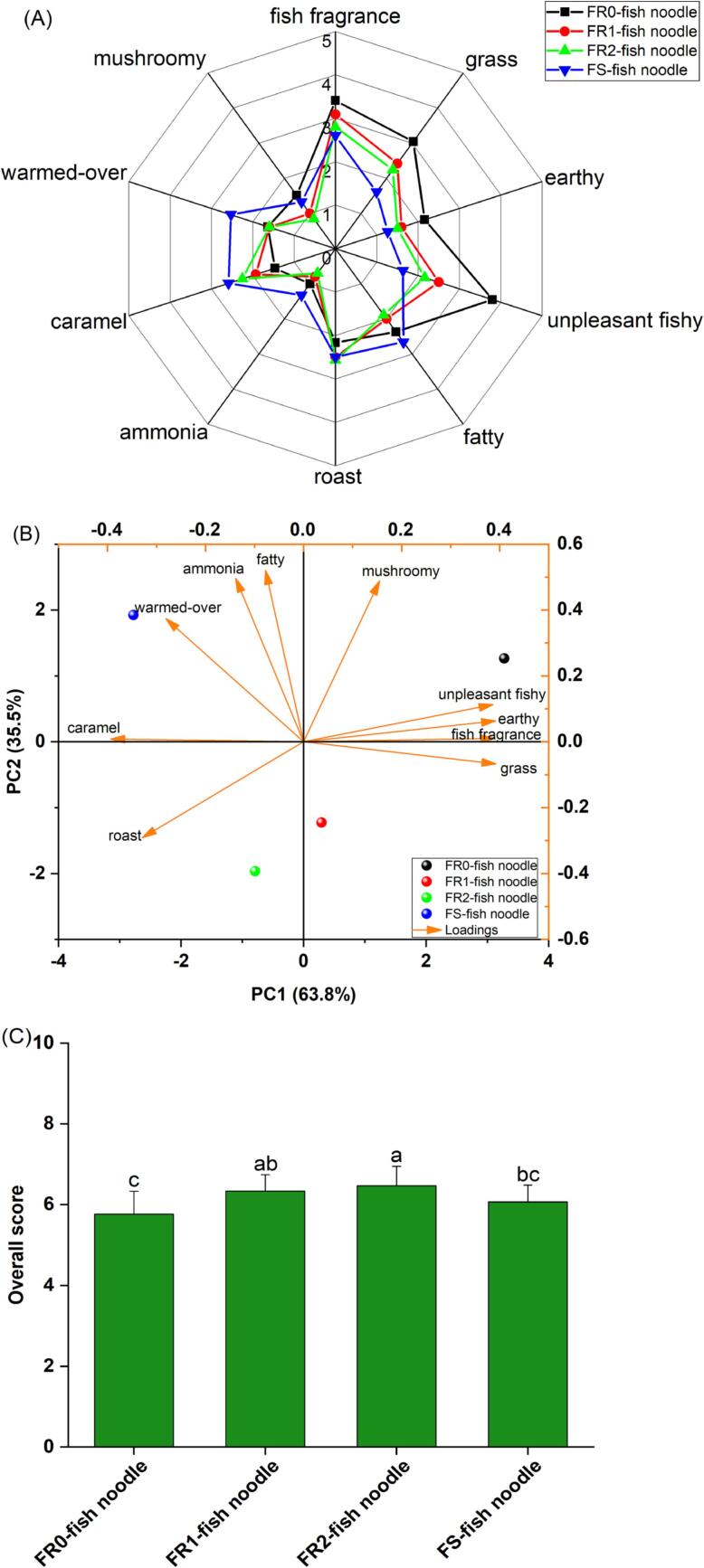
Overall odor rating of fish noodle. (A) Intensity values of the ten odor attributes. (B) Biplot plot of PCA based on odor intensity values of fish noodle. (C) Overall flavor score of each sample. “a,b,c,d” represent significant relationships, *p* < 0.05.

A total of 10 descriptors (“grass, fish fragrance, unpleasant fishy, fatty, roast, ammonia, caramel, warmed-over, earthy, and mushroomy”) were defined as fish noodle characteristic odor attributes. As depicted in Fig. 2(A), “fish fragrance”, “grass”, and “unpleasant fishy” showed higher odor intensity in the fish noodles made from fresh fish mince, and the overall odor intensity decreased in the FR-fish noodles as the number of rinses increased. Thus, rinsing reduced some odorants and odorant substance-related aroma precursors (Phetsang et al., 2021). Fish noodles from frozen surimi exhibited a stronger “fatty” and “warmed-over” odor, potentially related to more VCs generated by fat autoxidation and chemical volatilization of polyunsaturated fatty acids during the storage and transportation of frozen surimi. Sato et al. (1973) showed that “warmed-over” was mainly present in frozen, chilled, pre-cooked, and cooked meat products, which was consistent with the results of this study.

The intensity of odor attributes was analyzed using PCA. Fig. 2(B) presents the four groups of fish noodles in four quadrants, indicating that odor attributes could significantly distinguish different fish noodles. Fig. 2(C) highlights that FR2-fish noodles had the highest total score and attained the highest consumer acceptance. In summary, rinsing effectively improves the flavor quality of fish noodles, especially those made from fresh fish. Interestingly, although the strongest fish odor was found in FR0-fish noodles, the other samples had significantly higher total scores, which indicates that the intensity of undesirable odors (unpleasure fishy, fatty) tends to affect the overall product acceptance more easily.

### 3.2 Detected volatile compounds in fish noodles by HS-GC-IMS

**Fig. 3:**
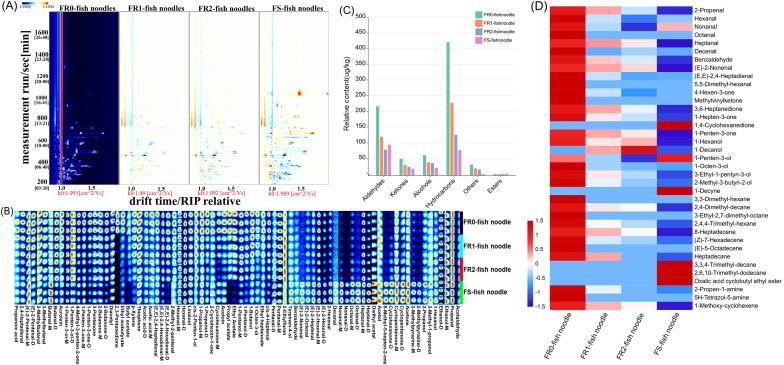
Comparison of volatile compounds in fish noodles by GC-IMS and GC-MS. (A) Two-dimensional differential map of HS-GC-IMS spectra obtained from fish noodles. (B) Fingerprints map of volatile compounds established by GC-IMS. (C) Relative content of volatile compound species in the fish noodles by GC-MS. (D) Fingerprint map of volatile compounds established by GC-MS.

VCs in fish noodles were analyzed using the HS-GC-IMS technique. In Fig. 3(A) (FR0), each point on either side of the reactive ion peak represents a VC. To compare the differences of volatile compounds in fish noodles during different rinsing processes, we used the FR0-fish noodle plot as a reference, and after deducting the reference plot in FR1, FR2, and FS plots, the bottom color of the plot changed to white. The blue area indicates that the VC concentration was lower in the sample than FR0, and the red area indicates that the VC concentration was higher than FR0. The darker the color, the greater the difference. As depicted in Fig. 3(A), VCs were mainly distributed in the retention time range of 200–1000 s and drift time of 1.0–1.5 s. Interestingly, the volatility profile of the FS-fish noodle showed a very different profile from the other three fresh groups, especially in the 1000–1200 retention time, where a brighter signal appeared. Indeed, the VCs of fish noodles made from fresh surimi and frozen surimi are very different, indicating that the freshness of raw materials affects the quality of fish noodles and consumers' acceptability. Thus, VCs can also be used to judge the freshness of raw materials for making fish noodles.

Table S1 (Supplementary Information) reports the VCs’ qualitative analysis, revealing that HS-GC-IMS detected 87 signal peaks in fish noodles, and 80 VCs were identified, while 7 peaks were not. Notably, 20 VCs existed as monomers (-M) and dimers (-D), and the formation of these monomers and dimers was intimately associated with the concentrations of VCs and their half-lives (Garrido-Delgado et al., 2015). Thus, 62 VCs were identified in fish noodles, including 25 aldehydes, 14 alcohols, 11 ketones, 5 esters, 2 acids, and 5 others. Since the GC-IMS system could not ionize and detect compounds with proton affinity less than water (691.0 kJ/mol), the alkanes had a proton affinity of 543.5 KJ/mol. Therefore, these could not be detected by GC-IMS (Yao et al., 2022). To further investigate the differences in VCs between fish noodles, using the signal intensity of VCs as a benchmark, the fingerprint profiles of four groups of fish noodles were constructed, as depicted in Fig. 3(B).

Aldehydes were identified as one of the principal VCs in aquatic products. In this study, hexanal, pentanal, heptanal, butanal-D, nonanal-M, acrolein, propanal, acetaldehyde, (E,E)-2,4-hexadienal-M, (E)-2-hexenal, and (E)-2-pentenal have high signal intensity in the fish noodles. The main sources of aldehydes were connected with unsaturated lipid oxidation and Strecker degradation of amino acids (Wu & Wang, 2019). As the number of rinses increased, the intensity of most aldehyde signals in fish noodles made from fresh fish mince showed different degrees of reduction, which agreed with the results by Li et al. (2023) that rinsing processing affects the chemical composition (such as free fatty acids, amino acids, and lipoxygenase) in fish mince and reduces the content of VCs in surimi products. In fish noodles made from frozen surimi, acrolein, propanal, butanal, pentanal, hexanal, and (E,E)-2,4-hexadienal have relatively low signal intensity, and interestingly the odor properties of these VCs were characterized as “fresh, grassy, and green”, whereas VCs with a typical odor described as “greasy, fatty”, e.g., heptanal, nonanal, (E)-2-heptenal, (E)-2-octenal, (E)-2-nonenal and (E)-2-decenal showed stronger signal intensity, consistent with the sensory evaluation of FS-fish noodle which showed a low intensity of grassy odor and high intensity of the fatty odor. In addition, many studies showed that aldehydes with lower odor thresholds and unique odor characteristics contributed significantly to the product aroma (Moretti et al., 2017).

Alcohols are mostly degradation products of fatty acid oxides or reduction products of carbonyl compounds and usually have an aromatic odor (Yao et al., 2022). In this study, the alcohols in fish noodles mainly included terpinen-4-ol, linalool, *cis*-2-penten-1-ol, 1-pentanol, 1-penten-3-ol, 1-butanol, 1-propanol, ethanol, 2-propanol, 1-octen-3-ol, and 1-hexanol, which agreed with other reports of VCs in fish products (Zhang et al., 2020). Due to their lower threshold, unsaturated alcohols contribute more to product odor. Besides, 1-Octen-3-ol is often considered to be the critical VC in raw fish, and the high volatility, which results in significant losses during thermal processing (Wei et al., 2023), corresponded to the low signal intensity of 1-octen-3-ol detected in this study. In addition, 1-penten-3-ol and *cis*-2-penten-1-ol have relatively low signal strength in fish noodles made from frozen surimi, and it has been suggested that they provide an unpleasant odor to aquatic products.

Ketones generally have a modifying effect on the product’s odor because of their high threshold. The thermal oxidation degradation of unsaturated fatty acids or amino acids is an important pathway for ketone generation. For example, 2,3-pentanedione, 1-penten-3-one, 3-pentanone, 2-butanone, and diacetyl, which have a “buttery, caramel” flavor, are present in high concentrations in FR0-fish noodles. The processing temperature of the product easily influences its concentrations (Yao et al., 2022). However, high levels of acetone in FS-fish noodles reflect the degree of decay of fish, while 6-methyl-5-hepten-2-one is generally produced by the auto-oxidation of linoleic acid and linolenic acid (Leduc et al., 2012, Wu et al., 2021).

Esters usually present fruity and floral flavors because of their low content and large odor threshold, contributing less to the odor of aquatic products. In this study, the detected esters in fish noodles include ethyl caproate, propyl acetate, ethyl acetate, isobutyl acetate, and butyl acetate. Compared with FR-fish noodle, the ester content in FS-fish noodles is relatively low, especially propyl acetate, which was almost undetectable.

Furthermore, some other compounds in this study are mostly related to the Maillard reaction. Acetic acid is the hallmark product of the 2,3-enolization pathway in the Maillard reaction (Wei et al., 2023). Additionally, 2-Ethylfuran has a sweet, meaty, and caramel odor and can be produced by thermal degradation of carbohydrates, the Maillard reaction, and lipid oxidation. Moreover, 2-Methylpyrazine has a very low threshold and is mainly produced through the condensation reaction of α-amino ketones in the Maillard reaction, with the highest content in FS-fish noodles.

### 3.3 Detected volatile compounds in fish noodles by HS-SPME-GC-MS

HS-SPME-GC-MS analyzed VCs in fish noodles, and the results are reported in Table S2 (Supplementary Information). A total of 37 VCs (10 aldehydes, 6 ketones, 6 alcohols, 1 ester, 11 hydrocarbons, and 3 others) were detected in the samples, which were significantly different from the results of HS-GC-IMS due to the different detection principles of the two methods. Li et al. (2022) determined the volatile compounds of Nanjing brine duck by GC-IMS and GC-MS and identified 50 and 31 VCs, respectively. In addition, different injection methods and differences in the ability of different columns to separate VCs are also important reasons for this result (Wang et al., 2023). The relative content of the 37 VCs in fish noodles was identified and used as a benchmark for the flavor heat map. Fig. 3(D) reveals that in FR-fish noodles, the amount of VCs in fish noodles decreases gradually as the number of rinsing increases. Hydrocarbons were the highest relative volatile substance in the fish noodles, but their threshold was high and usually considered not to affect food flavor.

Fig. 3(C & D) suggests that compared to the FR0-fish noodle, the relative contents of aldehydes, ketones, and alcohols decreased by 44.93 %, 59.58 %, and 40.11 %, respectively, in the FR1 group and by 63.99 %, 52.16 % and 42.28 %, in the FR2 group. Besides, VCs with higher contents in FR0-fish noodles were hexanal, nonanal, benzaldehyde, 1-penten-3-one, and 2-methyl-3-butyn-2-ol, which were also the major VCs in FR1 and FR2-fish noodles. Compared to FR-fish noodles, the VCs of FS-fish noodles differed significantly. FS-fish noodles showed a 56.92 %, 52.16 %, and 42.28 % decrease in aldehydes, ketones, and alcohols, respectively, compared to FR0, while FS-fish noodle samples showed a 20.19 % increase in aldehydes and a 26.42 % and 43.01 % decrease in ketones and alcohols, respectively, compared to FR2 samples. It should be noted that heptanal, 3,6-heptanedione, 1-hepten-3-one, 1-penten-3-one, 1-hexanol, 1-decanol, and 3-ethyl-1-pentyn-3-ol were not detected in the FS-fish noodle and 1,4-cyclohexanedione was only detected in the FS-fish noodle. This suggests that microbial metabolism and degradation of flavor precursors during the freezing of surimi may impact the alteration of VCs in fish noodles and affect the flavor characteristics of fish noodles.

### 3.4 Multivariate statistical analysis

#### 3.4.1 Analysis of potentially characteristic volatile compounds

**Fig. 4:**
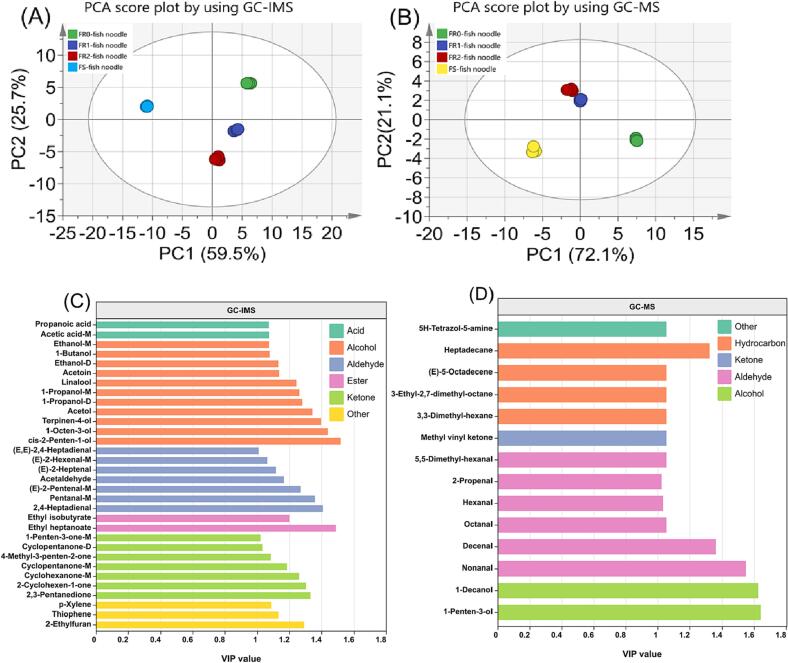
(A&B) PCA score plots of the volatile profiles detected by GC-IMS and GC-MS. (C&D) VIP scores of volatile compounds detected by GC-IMS and GC-MS in the PLS-DA model.

In order to better elucidate the reasons for the differences in aroma characteristics between fish noodle samples and to reveal the differences in VCs of the samples, the volatile profiles determined by GC-MS and GC-IMS were analyzed using unsupervised PCA and supervised PLS-DA. The PCA scores plots of GC-MS and GC-IMS are illustrated in Fig. 4(A & B), revealing that the cumulative discriminant indices of PCA were 85.2 % (Fig. 4A) and 93.2 % (Fig. 4B). Thus, both GC-IMS and GC-MS were effective in distinguishing the differences in VCs of different fish noodle samples. Meanwhile, the different groups of fish noodles demonstrated the same segregation trend in PCA (Fig. 4A & B) and PLS-DA scoring plots (Fig. S1A & B) (Supplementary Information). FR1 and FR2 fish noodles were close to each other and located in the same quadrant, while FR0 and FS fish noodles were located in the other different quadrants. This indicates that rinsing times had a minor effect on VCs in fresh fish noodles. However, there was a significant difference between rinsed and non-rinsed volatile compounds in fish noodles.

A substitution test was conducted to verify whether the PLS-DA model was qualified for subsequent screening of VCs, with the corresponding results depicted in Fig. S1(C & D). R2 represents the cumulative variance value, and Q2 represents the cumulative cross-validity, and when R2X-R2Y < 0.3 and Q2 > 0.5 in the model, it indicates a good model fit (Zhu et al., 2022). GC-IMS and GC-MS validated the model with R2X-R2Y at −0.067 and −0.018, and Q2 was 0.975 and 0.912, respectively, initially indicating that the PLS-DA models were relatively reliable. In addition, the experimental values of R2 and Q2 generated by random permutation in Fig. S1(C & D) were lower than the rightmost R2 and Q2 values, and the intercept of the Q2 regression line was negative, which indicated that both PLS-DA models were free of overfitting and could be directly used for subsequent discriminant analysis of VCs. The variable importance in the projection (VIP) of the GC-IMS model was calculated, in which VCs with VIP greater than 1 could be used as potential characteristic markers of the samples, and the larger the VIP value, the stronger the differentiation ability of the samples. As shown in Fig. 4(C), a total of 32 VCs (7 aldehydes, 11 alcohols, 7 ketones, 2 esters, 2 acids, and 3 other compounds) were identified as potential signature compounds for fish noodles, among which *cis*-2-penten-1-ol, ethyl heptanoate, 1-octen-3-ol, and 2, 4-heptadienal made the greatest. Similarly, Fig. 4(D) shows 15 potentially iconic VCs, 6 aldehydes, 2 alcohols, 1 ketone, 1 other, and 4 hydrocarbons, among which 1-penten-3-ol, 1-decanol, and nonanal are the most contributing compounds with VIP values greater than 1.5.

#### 3.4.2 PLSR analysis

**Fig. 5:**
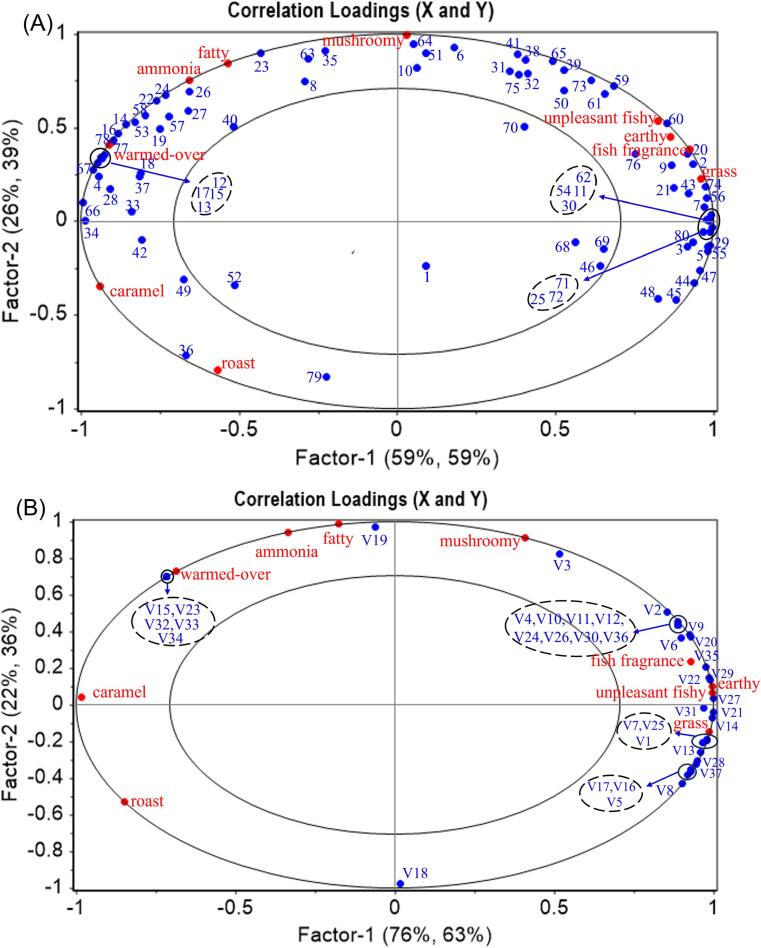
Correlation loading plot from PLSR. The ellipses represent R2 values of 0.5 and 1.0, with each ellipse corresponding to a specific R2 value. (A) X matrix is the signal intensity projection of volatile compounds identified by GC-IMS, and the Y matrix is the intensity projection of characteristic odor attributes. (B) X matrix is the relative content projection of volatile compounds identified by GC-MS, and the Y matrix is the intensity projection of characteristic odor attributes.

It is well known that food odor is closely related to VCs. Somjid et al. (2021) reported that a decrease in the relative content of aroma compounds may lead to a decreasing trend in the odor of surimi products. In addition, the effect of VCs in foods on the overall aroma is complex and may have synergistic or antagonistic effects with each other, and not all VCs contribute to the flavor of foods (Liu et al., 2023, Niu et al., 2020, Vilar et al., 2020). To further investigate the correlation between VCs and sensory attributes, two PLSR models were employed (Fig. 5).

Fig. 5(A) highlights that PC1 and PC2 explained 84 % of the cross-validation variance of the X variable and 98 % of the cross-validation variance of the Y variable. Additionally, all sensory attributes and most VCs were distributed between ellipses with R2 of 0.5 and 1, indicating that the model explained the correlation between sensory attributes and VCs (Nie et al., 2023). From the previous sensory evaluation results, “unpleasant fishy”, “fish fragrance”, and “grass” odors were the stronger characteristic flavors in the fish noodles, and they were located in the first quadrant in the loading diagram. The nearby VCs mainly included 1-penten-3-ol (60), (E)-2-pentenal-D (20), acrolein (2), 3-methylbutanal (9), hexanal-D (11), pentanal-D (7), and (E,E)-2,4-heptadienal-D (30), suggesting that these compounds may contribute significantly to the characteristic odor. In addition, the distribution of the “warmed-over” and “fatty” odors was in the second quadrant, where the compounds distributed near the “warmed-over” mainly included 2-methylpyrazine (77 & 78), heptanal (12 & 13), octanal (14 & 15), nonanal (16 &17), 6-methyl-5-hepten-2-one (67), suggesting that these compound contents were closely related to the “warmed-over” flavor in the fish noodles. This result is similar to previous studies in which An et al. (2022b) confirmed that (E,E)-2,4-decadienal, heptanal, octanal, nonanal, decanal, (E)-2-nonanal and 2-propylpyridine play an important role in contributing to the “warmed- over” flavor of surimi products by using aroma extraction dilution analysis, aroma recombination and omission studies.

The GC-MS model explained 98 % and 99 % of the cross-validation variance of the X and Y variables, respectively. Most of the VCs in Fig. 5(B) were clustered on the right side, and the sensory attributes distributed nearby were “grass”, “fish fragrance”, and “unpleasant fishy”, which indicated that these three odors might be the main odors of the fish noodle samples, consistent with the previous sensory evaluation results. Additionally, compared with the PLSR model of GC-MS, the GC-IMS model revealed the association of each odor with VCs in fish noodles more comprehensively and was more suitable for exploring the key characteristic aroma compounds.

### 3.5 Potential transformation pathways of VCs in fish noodles

**Fig. 6:**
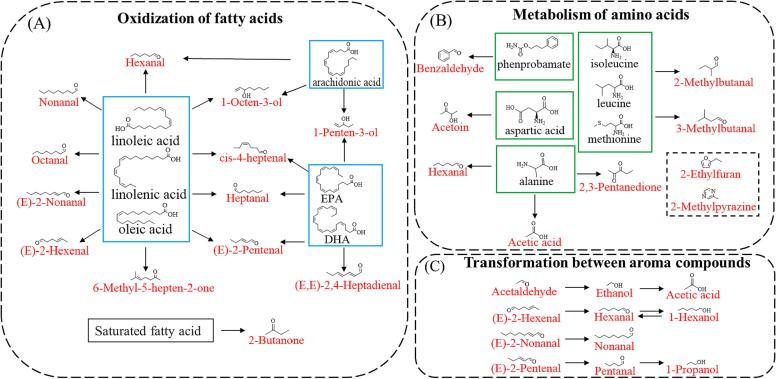
Analysis chart of potential sources of volatile compounds. (A) Oxidization of fatty acids. (B)Strecker pathway and Maillard reaction. (C) Transformation between aroma compounds.

In the previous section, we discussed the production pathways of a few compounds in fish noodles. To further understand the mechanism of aroma formation, possible precursors and metabolic processes of some fish noodle aroma compounds were predicted using literature reports as well as the KEGG platform (Fig. 6). As depicted in Fig. 6(A), fatty oxidation is one of the important sources of VCs, among which hexanal, octanal, decanal, nonanal, (E)-2-hexenal, *cis*-4-heptenal, (E)-2-heptenal, (E)-2-nonenal and 1-octen-3-ol can be generated by the decomposition of monounsaturated fatty acids (oleic acid, linoleic acid, linolenic acid) (Bassam et al., 2022, Li et al., 2020, Mahmoud and Buettner, 2017, Wu et al., 2021). The 1-Penten-3-ol can be produced by the degradation of polyunsaturated fatty acids (EPA, arachidonic acid), and arachidonic acid also produces hexanal under high-temperature aerobic conditions, while heptanal is also a product of oxidative degradation of DHA (Liu et al., 2022, Wu et al., 2021). The pathways for forming these compounds are similar, following a free radical oxidation mechanism that arises mainly from lipids' auto-oxidation and thermal oxidation. Fatty acids form hydroperoxides by removing hydrogen radicals from alkyl groups, adding O_2_, and uptake of hydrogen radicals, which then split, forming volatile compounds. In addition, lipid oxygenase catalyzing the breakdown of hydroperoxides may also be a pathway for producing volatile compounds on fish noodles.

Fig. 6(B) presents another important pathway for compound generation in fish noodles: the metabolism of amino acids. The benzaldehyde is a degradation product of phenylalanine, 2/3-methylbutanal are Strecker degradation products of isoleucine, leucine, methionine, and alanine can be degraded to acetic acid, hexanal, and 2,3-pentanedione. Similarly, none of these VCs can be produced without the catalysis of high-temperature conditions. However, acetylene is different in that it can be produced from aspartic acid by a transamination reaction (Bassam et al., 2022, Sriphochanart and Skolpap, 2018). In addition to the above two pathways, interconversion of compounds is also one of the important pathways for generating compounds in the product. As shown in Fig. 6(C), unsaturated aldehydes undergo chemical reactions under the synergistic action of heat and oxygen to produce the corresponding saturated aldehydes, and the resulting aldehydes can undergo further reactions to convert to alcohols or acids (Al-Dalali et al., 2022). In addition, inverse hydroxyl aldol condensation is also an important pathway for generating compounds in surimi products. It was found that the conversion of *cis*-4-heptenal to (E,Z)-2,6-nonadienal and the conversion of (E)-2-pentenal to (E,Z)-2,4-heptadienal in surimi products of silver carp (An et al., 2022b), unfortunately, (E,Z)-2,6-nonadienal and (E,Z)-2,4-heptadienal were not detected in this study in fish noodles, 2,4-heptadienal, both of which were detected in the present study.

## 4 Conclusions

This study investigated the effects of different fresh and frozen surimi compositions on the aroma quality of fish noodles. Ten odor attributes were screened by sensory evaluation as characteristic odor attributes of fish noodles, among which FR0-fish noodle samples had the strongest “fish fragrance” and “unpleasant fishy” flavor, FS-fish noodle samples had the strongest “warmed-over” and “fatty” flavor. In contrast, FR2-fish noodles had the highest overall score. A combination of HS-SPME-GC-MS and HS-GC-IMS was used to obtain the fish noodle aroma fingerprints, which could visualize the differences of the VCs in each group of samples, in which the aroma characteristics of FR and FS fish noodles differed significantly, and the aroma compounds of FR1 and FR2 fish noodle were similar. In addition, GC-IMS and GC-MS screened 32 and 15 compounds that could well reflect the differences in aroma components among fish noodles as potential signature compounds of fish noodles by VIP values, respectively. Finally, the correlation analysis revealed that the content of compounds such as 1-penten-3-ol, (E)-2-pentenal-D, acrolein, and 3-methylbutanal closely related to the “fish fragrance” and “unpleasant fishy” flavor and the content of compounds such as 2-methylpyrazine, heptanal, octanal, nonanal were closely related to “warmed-over” flavor. The results of this study can provide theoretical guidance for the quality improvement and standardization of fish noodles.

## CRediT authorship contribution statement

**Hongyu Zhou:** Conceptualization, Writing – original draft. **Zhiwei Hu:** Methodology, Investigation. **Youming Liu:** Writing – review & editing, Supervision. **Shanbai Xiong:** Project administration, Funding acquisition.

## Declaration of Competing Interest

The authors declare that they have no known competing financial interests or personal relationships that could have appeared to influence the work reported in this paper.

## Data Availability

No data was used for the research described in the article.
