# Supplementary material for: Flavor and sensory profile of Chinese traditional fish noodles produced by different silver carp (hypophthalmichthys molitrix) mince ingredients
Source: Food Chem X. 2023 Nov 1;20:100977. doi: 10.1016/j.fochx.2023.100977 (PMC10740137; doi:10.1016/j.fochx.2023.100977)
Supplement: Supplementary data 1 [file mmc1.docx]

**Figure S1 PLS-DA** **analytical model.**

**
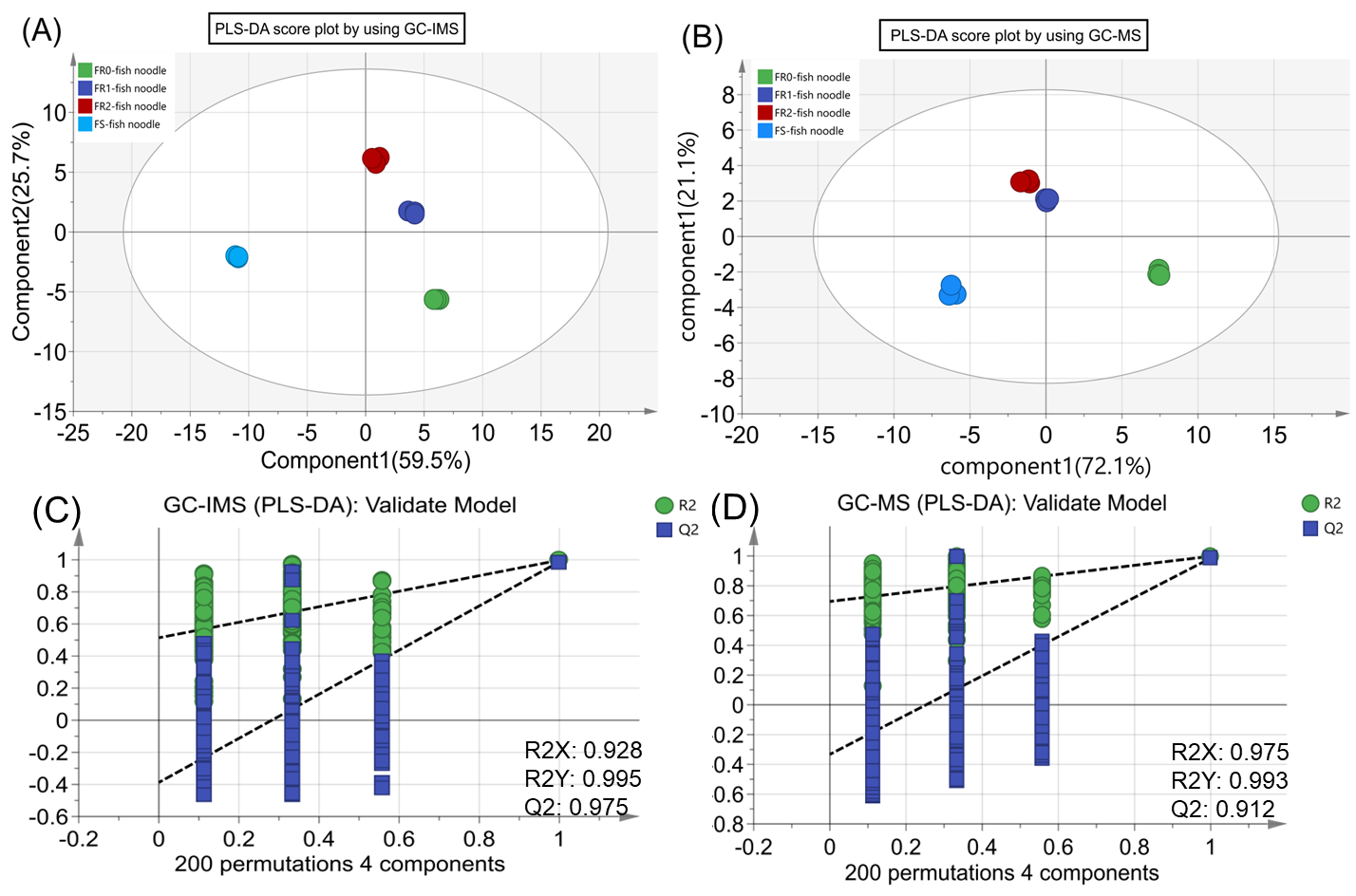
**

**Table S1 Identification volatile compounds in four groups of fish noodle using GC-IMS**

| No. | Compound | RI^a^ | Rt ^b^ [sec] | Dt^c^ [RIPrel] | Peak intensity of fish noodle | | | | odor description^d^ |
| --- | --- | --- | --- | --- | --- | --- | --- | --- | --- |
|  |  |  |  |  | FR0 | FR1 | FR2 | FS |  |
|  | *Aldehydes(34)* |  |  |  |  |  |  |  |  |
| 1 | Acetaldehyde | 780.6 | 219.064 | 0.98049 | 1808.47±41.08 | 1977.25±85.12 | 1858.26±211.50 | 1837.13±216.73 | green apple |
| 2 | Acrolein | 878.3 | 273.035 | 1.0587 | 2451.99±25.47 | 2222.43±30.02 | 1870.82±55.38 | 1647.54±73.24 |  |
| 3 | Propanal | 831.3 | 245.576 | 1.1423 | 10636.67±127.82 | 10626.39±154.72 | 10431.87±150.41 | 9925.46±124.62 | caramel |
| 4 | Butanal-M | 902.3 | 288.185 | 1.11129 | 1830.84±12.25 | 1776.73±8.03 | 1898.02±28.87 | 2130.94±1.91 | smoky, fish |
| 5 | Butanal-D | 902.3 | 288.185 | 1.28794 | 5413.83±41.44 | 5330.14±38.44 | 4849.62±82.53 | 3340.75±129.84 | smoky, fish |
| 6 | Pentanal-M | 1007.7 | 368.427 | 1.178 | 2202.49±19.75 | 1894.07±38.35 | 1870.71±13.35 | 2040.24±19.68 | green |
| 7 | Pentanal-D | 1009 | 369.951 | 1.42461 | 5182.79±38.21 | 5266.47±16.79 | 4459.13±14.06 | 3783.77±74.94 | green |
| 8 | 2-Methylbutanal | 940 | 313.751 | 1.16253 | 447.58±35.76 | 413.26±8.26 | 398.2±11.52 | 460.11±25.46 | malty |
| 9 | 3-Methylbutanal | 941.4 | 314.697 | 1.40256 | 220.27±13.91 | 195.71±3.87 | 169.82±18.58 | 149.96±17.03 | malty |
| 10 | Hexanal-M | 1105.8 | 494.381 | 1.26023 | 5295.11±43.74 | 5220.62±45.96 | 5189.82±28.80 | 5264.07±16.03 | grassy |
| 11 | Hexanal-D | 1106.9 | 496.202 | 1.56427 | 14255.02±86.80 | 13872.46±94.20 | 13516.76±62.87 | 12466.89±186.83 | grassy |
| 12 | Heptanal-M | 1202.2 | 672.229 | 1.32856 | 3726.28±55.16 | 3605.83±20.48 | 3783.76±85.04 | 4744.94±44.13 | creamy |
| 13 | Heptanal-D | 1202.2 | 672.229 | 1.69647 | 2420.57±64.13 | 2319.91±31.05 | 2766.56±72.54 | 5944.44±133.84 | creamy |
| 14 | Octanal-M | 1301.1 | 825.445 | 1.40426 | 398.34±15.30 | 406.33±15.58 | 516.81±9.87 | 1101.35±25.59 | citrus, fatty |
| 15 | Octanal-D | 1301.3 | 825.855 | 1.82963 | 49.91±1.08 | 51.89±2.03 | 53.46±8.35 | 115.42±4.74 | citrus, fatty |
| 16 | Nonanal-M | 1406.3 | 1037.127 | 1.48072 | 1097.06±36.30 | 1168.52±12.39 | 1550.41±22.72 | 5125.96±133.28 | citrus, fatty |
| 17 | Nonanal-D | 1406.4 | 1037.296 | 1.95124 | 268.61±12.57 | 325.58±74.31 | 337.30±34.15 | 1717.91±51.75 | citrus, fatty |
| 18 | Decanal | 1530.5 | 1358.036 | 1.549 | 211.24±42.00 | 221.46±24.67 | 220.25±39.13 | 333.14±37.98 | fatty,burnt |
| 19 | (E)-2-Pentenal-M | 1157.3 | 586.529 | 1.10775 | 2853.68±62.99 | 2602.32±96.30 | 2789.8±23.56 | 3139.52±42.40 | green |
| 20 | (E)-2-Pentenal-D | 1157 | 585.943 | 1.36346 | 4067.74±104.83 | 3515.97±129.92 | 2914.92±77.50 | 2541.92±40.73 | green |
| 21 | 2-Methyl-2-pentenal | 1172.6 | 616.999 | 1.1606 | 188.81±8.69 | 174.81±10.07 | 168.42±4.84 | 151.92±6.60 |  |
| 22 | 2-Hexenal | 1219.7 | 697 | 1.18422 | 224.47±4.66 | 187.74±1.53 | 168.49±7.93 | 336.85±5.48 |  |
| 23 | (E)-2-Hexenal-M | 1236.6 | 721.772 | 1.18246 | 2910.76±39.11 | 2513.54±60.73 | 2209.26±9.35 | 3182.81±11.39 | nutty |
| 24 | (E)-2-Hexenal-D | 1237.2 | 722.689 | 1.52044 | 1451.70±45.71 | 1141.69±21.61 | 915.52±39.74 | 2359.94±39.94 | nutty |
| 25 | cis-4-Heptenal | 1259.4 | 756.635 | 1.15605 | 415.97±8.25 | 406.73±33.69 | 341.69±8.12 | 228.6±10.30 | fishy |
| 26 | (E)-2-Heptenal | 1333.7 | 885.997 | 1.26167 | 269.03±7.42 | 256.91±3.66 | 210.4192±10.49 | 347.95±8.35 | fatty |
| 27 | (E)-2-Octenal | 1441.2 | 1118.676 | 1.33978 | 255.04±35.11 | 213.14±14.88 | 213.32±5.61 | 328.04±48.88 | oily, nutty |
| 28 | (E)-2-Nonenal | 1573.7 | 1491.556 | 1.41914 | 258.74±29.32 | 258.39±49.83 | 310.88±57.83 | 458.69±10.50 | oily, creamy |
| 29 | (E,E)-2,4-Hexadienal-M | 1353.8 | 925.448 | 1.105 | 2571.36±26.21 | 2546.91±78.38 | 2188.36±23.68 | 1326.22±83.70 | green, spicy |
| 30 | (E,E)-2,4-Hexadienal-D | 1354.2 | 926.365 | 1.45354 | 300.93±8.95 | 273.97±28.42 | 215.43±5.04 | 102.02±11.36 | green, spicy |
| 31 | 2,4-Heptadienal | 1491.1 | 1246.596 | 1.20347 | 491.37±17.74 | 456.58±10.70 | 352.16±8.78 | 433.69±24.63 |  |
| 32 | (E,E)-2,4-Heptadienal | 1520.8 | 1329.744 | 1.19423 | 586.31±11.93 | 509.17±31.62 | 477.21±28.49 | 516.12±27.05 | fatty, fishy |
| 33 | Benzaldehyde | 1550.3 | 1417.539 | 1.16259 | 233.18±17.95 | 253.37±20.43 | 254.19±19.93 | 300.88±15.74 | green, rosy |
| 34 | Diethyl acetal | 918 | 298.575 | 1.02838 | 562.08±10.48 | 732.29±4.72 | 771.10±38.38 | 1127.40±29.46 |  |
|  | *Alcohols (18)* |  |  |  |  |  |  |  |  |
| 35 | Ethanol-M | 953.2 | 323.219 | 1.04387 | 2574.37±16.28 | 2335.82±45.60 | 2271.53±111.53 | 2579.42±44.30 | alcohol |
| 36 | Ethanol-D | 953.9 | 323.693 | 1.12612 | 10405.12±28.91 | 11309.67±16.58 | 11577.33±130.96 | 11480.84±31.77 | alcohol |
| 37 | 2-Propanol | 944.7 | 317.065 | 1.08837 | 279.09±10.51 | 263.29±11.44 | 291.16±9.38 | 344.10±38.03 | fruity, fresh |
| 38 | 1-Propanol-M | 1060.6 | 431.1 | 1.11338 | 1841.82±51.58 | 1508.32±15.01 | 1450.15±39.99 | 1578.60±36.33 |  |
| 39 | 1-Propanol-D | 1060.2 | 430.545 | 1.24684 | 809.39±9.48 | 502.24±10.30 | 455.26±4.73 | 524.01±18.53 |  |
| 40 | Acetol | 1317.2 | 854.803 | 1.05395 | 110.08±8.21 | 114.42±5.09 | 99.64±3.87 | 126.38±10.84 |  |
| 41 | Acetoin | 1302.1 | 827.279 | 1.06099 | 580.23±8.84 | 392.13±20.58 | 311.96±42.24 | 424.68±21.59 | butter |
| 42 | 2-Methyl-1-propanol | 1113.3 | 506.937 | 1.17426 | 96.51±8.61 | 100.75±4.72 | 107.45±6.27 | 116.60±6.16 |  |
| 43 | 1-Butanol | 1165.6 | 602.936 | 1.18346 | 545.72±14.64 | 549.74±6.42 | 455.18±15.96 | 404.78±13.77 |  |
| 44 | 1-Penten-3-ol-M | 1180.2 | 632.82 | 0.94346 | 4886.95±26.37 | 4967.54±65.82 | 4809.99±46.36 | 3993.32±33.67 | mushroom |
| 45 | 1-Penten-3-ol-D | 1180.7 | 633.992 | 1.31203 | 1271.40±24.39 | 1344.41±30.79 | 1288.03±112.37 | 812.14±32.17 | mushroom |
| 46 | cis-2-Penten-1-ol | 1339.9 | 897.924 | 0.94657 | 655.69±43.95 | 645.75±27.98 | 571.03±11.21 | 511.48±61.33 | chemical, synthetic |
| 47 | 1-Pentanol-M | 1268.1 | 770.397 | 1.25815 | 864.68±31.79 | 835.82±13.66 | 815.08±19.67 | 500.88±18.10 | green,musty |
| 48 | 1-Pentanol-D | 1268.6 | 771.315 | 1.51164 | 124.48±14.25 | 139.89±20.82 | 128.28±7.45 | 68.66±10.71 | green,musty |
| 49 | 1-Hexanol | 1374.1 | 967.172 | 1.33326 | 123.48±25.27 | 138.67±11.51 | 159.15±10.33 | 166.32±19.31 | alcohol |
| 50 | 1-Octen-3-ol | 1487.9 | 1238.029 | 1.17249 | 287.71±6.90 | 266.17±9.74 | 182.12±5.22 | 226.78±19.37 | mushroom |
| 51 | Linalool | 1563.1 | 1457.664 | 1.23119 | 1159.04±36.83 | 938.03±59.36 | 1073.96±9.95 | 1111.49±39.39 | lavender |
| 52 | Terpinen-4-ol | 1634.6 | 1702.311 | 1.22888 | 411.00±18.58 | 511.21±76.79 | 475.68±20.25 | 511.86±27.55 | earthy |
|  | *Ketones (15)* |  |  |  |  |  |  |  |  |
| 53 | Acetone | 853 | 257.885 | 1.11803 | 5942.45±85.21 | 5715.84±40.39 | 5653.83±138.65 | 7137.69±206.27 |  |
| 54 | 2-Butanone-M | 928.5 | 305.702 | 1.0614 | 1442.16±8.67 | 1277.05±16.78 | 1265.12±28.00 | 957.01±4.30 | sweet |
| 55 | 2-Butanone-D | 927.8 | 305.229 | 1.25018 | 2139.29±34.33 | 1983.62±22.49 | 1736.26±24.53 | 753.64±64.44 | sweet |
| 56 | Diacetyl | 1002.3 | 362.647 | 1.1697 | 2024.00±44.75 | 1872.09±39.60 | 1754.07±43.63 | 1488.54±38.27 | butter |
| 57 | Cyclopentanone-M | 1148.5 | 569.656 | 1.11129 | 1634.57±31.40 | 1389.71±56.45 | 1537.13±101.64 | 1898.16±31.75 | mint |
| 58 | Cyclopentanone-D | 1149.8 | 572.084 | 1.3278 | 2802.80±115.44 | 1951.26±44.61 | 2388.95±38.47 | 4186.55±17.79 | mint |
| 59 | 1-Penten-3-one-M | 1047.4 | 414.461 | 1.07825 | 1688.78±15.57 | 1393.54±5.31 | 1149.57±13.81 | 1242.76±33.41 | paint-like |
| 60 | 1-Penten-3-one-D | 1047.8 | 415.016 | 1.31146 | 1260.85±27.16 | 898.74±8.19 | 644.22±9.20 | 563.53±22.17 | paint-like |
| 61 | 2,3-Pentanedione | 1087.2 | 466.456 | 1.22799 | 2753.91±160.45 | 258.88±58.14 | 125.03±31.60 | 74.60±10.49 | buttery |
| 62 | 3-Pentanone | 1006.3 | 366.979 | 1.34776 | 1982.74±21.40 | 1797.78±24.41 | 1714.05±10.84 | 1325.72±16.89 |  |
| 63 | 2-Cyclohexen-1-one | 1417.7 | 1063.067 | 1.12436 | 245.15±4.14 | 235.87±6.84 | 211.96±4.33 | 253.84±4.75 | fresh, sweet |
| 64 | Cyclohexanone-M | 1301.1 | 825.445 | 1.16485 | 521.74±40.18 | 387.94±25.57 | 187.10±7.60 | 461.55±32.87 | sweet, wine-like |
| 65 | 4-Methyl-3-penten-2-one | 1133 | 541.124 | 1.11436 | 1031.21±2.92 | 820.60±6.99 | 652.55±19.27 | 794.88±47.10 | sweet, chemical |
| 66 | Cyclohexanone-D | 1301.3 | 825.855 | 1.4659 | 132.75±17.50 | 200.14±19.10 | 274.97±12.44 | 584.03±10.77 | fresh, sweet |
| 67 | 6-Methyl-5-hepten-2-one | 1350 | 917.925 | 1.18053 | 101.39±6.57 | 118.04±10.31 | 157.01±9.51 | 517.37±18.59 | fruity |
|  | *Acids (3)* |  |  |  |  |  |  |  |  |
| 68 | Acetic acid-M | 1505 | 1284.972 | 1.06022 | 2521.61±87.52 | 2612.09±125.28 | 2596.75±144.21 | 2426.36±38.96 | vinegar |
| 69 | Acetic acid-D | 1505.6 | 1286.571 | 1.15495 | 147.02±15.11 | 154.84±1.35 | 152.31±19.10 | 128.81±7.56 | vinegar |
| 70 | Propanoic acid | 1639.4 | 1719.9 | 1.11336 | 580.53±64.71 | 556.82±48.90 | 486.44±25.90 | 521.57±8.33 |  |
|  | *esters(5)* |  |  |  |  |  |  |  |  |
| 71 | Ethyl acetate | 908.8 | 292.446 | 1.34053 | 643.84±23.58 | 545.53±20.21 | 452.60±11.90 | 131.37±10.48 | solvent, sweet |
| 72 | Propyl acetate | 1000.8 | 361.033 | 1.47897 | 966.42±38.22 | 736.35±17.26 | 674.50±7.82 | 75.15±5.63 |  |
| 73 | Ethyl isobutyrate | 975.4 | 339.789 | 1.18815 | 316.44±9.68 | 178.20±7.49 | 149.15±2.53 | 168.52±11.03 |  |
| 74 | Butyl acetate | 1096.5 | 479.394 | 1.23148 | 1072.79±18.39 | 750.26±29.63 | 621.81±29.52 | 227.94±14.16 |  |
| 75 | Ethyl heptanoate | 1343.6 | 905.264 | 1.40778 | 300.53±22.57 | 270.81±7.18 | 222.12±9.85 | 238.19±11.83 |  |
|  | *Others(5)* |  |  |  |  |  |  |  |  |
| 76 | Thiophene | 1069.2 | 442.193 | 1.04032 | 1834.23±55.82 | 1802.77±21.90 | 1680.80±72.75 | 1667.90±30.11 |  |
| 77 | 2-Methylpyrazine-M | 1239 | 725.442 | 1.06803 | 182.51±4.90 | 157.10±4.83 | 170.27±5.62 | 456.53±27.18 |  |
| 78 | 2-Methylpyrazine-D | 1238.9 | 725.22 | 1.4036 | 220.04±12.16 | 189.05±10.88 | 186.10±9.28 | 671.13±20.44 |  |
| 79 | 2-Ethylfuran | 981.6 | 344.524 | 1.04656 | 1497.54±17.28 | 1710.64±27.54 | 1719.24±69.75 | 1624.09±73.00 | fruity, floral |
| 80 | p-Xylene | 1151.5 | 575.396 | 1.07775 | 522.17±7.99 | 571.55±16.69 | 410.40±36.77 | 242.41±10.22 | Bitter almond |

Notes: a Retention index; b Retention time in the capillary GC column; c The drift time in the drift tube. d Odor description obtained from <http://www.thegoodscentscompany.com/index.html>. FR-fish noodle represented fish noodles made from fresh fish mince after rinsing. FR0, FR1, and FR2 represented the fresh fish mince with 0, 1, and 2 rinsing times, respectively; FS-fish noodle represented fish noodles made from frozen surimi.

**Table S2 Identification volatile compounds in four groups of fish noodle using GC-MS**

| NO. | Compounds | RI | Identification^a^ | Estimated concentration (μg/kg ) | | | |
| --- | --- | --- | --- | --- | --- | --- | --- |
|  |  |  |  | FR0 | FR1 | FR2 | FS |
|  | *Aldehydes(10)* | |  |  |  |  |  |
| V1 | Acrolein | - | MS | 2.57±0.19 | 1.75±0.04 | 1.14±0.32 | ND |
| V2 | Hexanal | 788 | RI MS STD | 108.76±10.8 | 55.24±11.48 | 32.76±5.72 | 48.05±3.91 |
| V3 | Nonanal | 1073 | RI MS STD | 43.26±2.7 | 24.16±3.54 | 11.13±1.96 | 31.5±1 |
| V4 | Octanal | 998 | RI MS STD | 1.41±0.02 | ND | ND | ND |
| V5 | Heptanal | 922 | RI MS | 13.52±0.55 | 10.12±0.16 | 8.36±0.04 | ND |
| V6 | Decenal | 1403 | RI MS | 3.76±0.29 | ND | 0.45±0.41 | ND |
| V7 | Benzaldehyde | 960 | RI MS | 18.15±3.47 | 13.05±1.08 | 11.17±2.98 | 5.15±0.23 |
| V8 | (E)-2-Nonenal | 1065 | RI MS STD | 9.26±0.12 | 7.55±0.83 | 7.06±0.84 | 2.37±1.2 |
| V9 | (E,E)-2,4-Heptadienal | 1015 | RI MS STD | 12.54±1.23 | 7.13±0.09 | 5.73±1.65 | 5.91±0.23 |
| V10 | 5,5-Dimethyl-hexanal | 1030 | RI MS | 2.81±0.85 | ND | ND | ND |
|  | *Ketones (6)* | |  |  |  |  |  |
| V11 | 4-Hexen-3-one | 931 | RI MS | 5.63±0.53 | 2.02±0.76 | 0.74±0.03 | 1.38±0.63 |
| V12 | Methylvinylketone | 518 | RI MS | 2.03±0 | ND | ND | ND |
| V13 | 3,6-Heptanedione | 832 | RI MS | 4.68±0.17 | 3.16±0.02 | 2.36±0.2 | ND |
| V14 | 1-Hepten-3-one | 892 | RI MS | 9.36±1.07 | 4.86±0.63 | 3.56±0.2 | ND |
| V15 | 1,4-Cyclohexanedione | 833 | RI MS | ND | ND | ND | 15.69±0.08 |
| V16 | 1-Penten-3-one | 651 | RI MS | 26.8±4.57 | 19.01±2.28 | 16.53±2.35 | ND |
|  | *Alcohols (6)* | |  |  |  |  |  |
| V17 | 1-Hexanol | 872 | RI MS STD | 7.63±0.6 | 4.62±2.22 | 5.12±0.96 | ND |
| V18 | 1-Decanol | 1443 | RI MS STD | 1.6±0.1 | 4.7±0.2 | 7.7±0.45 | ND |
| V19 | 1-Penten-3-ol | 688 | RI MS STD | 11.14±0.51 | 9.21±1.5 | 7.18±1.09 | 12.46±3.08 |
| V20 | 1-Octen-3-ol | 982 | RI MS STD | 6.57±0.32 | 3.46±0.27 | 3.12±0.04 | 3±0.49 |
| V21 | 3-Ethyl-1-pentyn-3-ol | 944 | RI MS | 3.2±0.44 | 1.58±0.2 | 1.15±0.05 | ND |
| V22 | 2-Methyl-3-butyn-2-ol | 781 | RI MS | 30.15±6.05 | 13.29±0.08 | 10.53±3.28 | 4.37±0.18 |
|  | *Hydrocarbons (11)* | |  |  |  |  |  |
| V23 | 1-Decyne | 1006 | RI MS | ND | ND | ND | 17±0.66 |
| V24 | 3,3-Dimethyl-hexane | 811 | RI MS | 13.2±2.03 | ND | ND | ND |
| V25 | 2,4-Dimethyl-decane | 1213 | RI MS | 33.5±15.22 | 18.29±3.32 | 16.93±2.98 | ND |
| V26 | 3-Ethyl-2,7-dimethyl-octane | 1205 | RI MS | 23.4±5.61 | ND | ND | ND |
| V27 | 2,4,4-Trimethyl-hexane | 901 | RI MS | 26.9±6.26 | 11.5±1.55 | 8.39±2.53 | ND |
| V28 | 8-Heptadecene | 1701 | RI MS | 7.61±0.81 | 5.18±0.69 | 4.26±1.96 | ND |
| V29 | (Z)-7-Hexadecene | 1603 | RI MS | 12.74±0.89 | 4.03±1.03 | 3.18±0 | ND |
| V30 | (E)-5-Octadecene | 1806 | RI MS | 10.71±0 | ND | ND | ND |
| V31 | Heptadecane | 1701 | RI MS | 292.22±99.91 | 187.95±39.47 | 92.47±35.9 | 24.89±8.03 |
| V32 | 3,3,4-Trimethyl-decane | 1311 | RI MS | ND | ND | ND | 25.4±2.29 |
| V33 | 2,6,10-Trimethyl-dodecane | 1503 | RI MS | ND | ND | ND | 9.31±0.85 |
|  | *Esters(1)* |  |  |  |  |  |  |
| V34 | Oxalic acid cyclobutyl ethyl ester | 1051 | RI MS | ND | ND | ND | 0.87±0.13 |
|  | *Others(3)* |  |  |  |  |  |  |
| V35 | 2-Propen-1-amine | - | MS | 6.52±0.96 | 2.14±0.49 | 0.98±0.46 | ND |
| V36 | 5H-Tetrazol-5-amine | - | MS | 0.41±0.42 | ND | ND | ND |
| V37 | 1-Methoxy-cyclohexene | 917 | RI MS | 23.07±2.26 | 15.88±1.18 | 13.33±1.91 | ND |

Notes: a MS is mass spectrometry identification, RI is retention index identification, and STD is standard validation. FR-fish noodle represented fish noodles made from fresh fish mince after rinsing. FR0, FR1, and FR2 represented the fresh fish mince with 0, 1, and 2 rinsing times, respectively; FS-fish noodle represented fish noodles made from frozen surimi.“ND” indicated “not detected”.
